# Effective Treatment of Folliculitis Decalvans With a Combination of Oral Isotretinoin and Rifampicin

**DOI:** 10.7759/cureus.60633

**Published:** 2024-05-19

**Authors:** Sugat Jawade, Sudhir Singh, Sabiha Quazi, Mayur Dudhe, Sabha Neazee, Soham R Meghe, Swapna Jawade

**Affiliations:** 1 Dermatology, Venereology, and Leprosy, Datta Meghe Medical College, Datta Meghe Institute of Higher Education and Research, Nagpur, IND; 2 Dermatology, Venereology, and Leprosy, Datta Meghe Medical College, Datta Meghe Institute of higher education and research, Nagpur, IND; 3 Dermatology, Venereology, and Leprosy, Jawaharlal Nehru Medical College, Datta Meghe Institute of Higher Education and Research, Wardha, IND; 4 Musculoskeletal Physiotherapy, Ravi Nair Physiotherapy College, Datta Meghe Institute of Higher Education and Research, Wardha, IND

**Keywords:** alopecia, cicatricial alopecia, combination therapy of isotretinoin and rifampicin, rifampicin, isotretinoin, folliculits decalvans

## Abstract

Folliculitis decalvans (FD) is a rare type of inflammatory scalp disorder that leads to scarring alopecia. It is classified as primary neutrophilic cicatricial alopecia. FD presents a challenging scenario in clinical dermatology due to its rarity, resistance to treatment, and potential for scarring alopecia. This inflammatory scalp disorder primarily affects middle-aged adults, predominantly males. While its exact pathogenesis remains uncertain, a deficient host immune response to Staphylococcus aureus infection is hypothesized. Therapeutic interventions for FD pose difficulties, with limited treatment options available

A 58-year-old female patient presented with a history of follicular papules that gradually progressed to form clusters of pustules, crusting, and hemorrhagic lesions with tufting of hairs on the crown area of the scalp, and was diagnosed with FD. Considering isotretinoin's role in inhibiting abnormal keratinization and inflammation, and rifampicin's ability to eradicate S. aureus, the combination of both provides a comprehensive approach to tackling the underlying factors contributing to FD. Despite previous unsuccessful treatments, combination therapy with isotretinoin and rifampicin yielded a remarkable outcome, prompting further exploration of this approach.

## Introduction

Quinquaud was the first to describe Folliculitis decalvans (FD) in 1988 by reporting a case of “folliculite épilante et destructive des regions velues” [1]. In 1905, Brocq et al. delineated Quinquaud's clinical observations, labeling them as *folliculitis decalvans* and differentiating this condition from other forms of cicatricial alopecia [2]. The incidence of folliculitis accounts for approximately 11% of all cases of cicatricial alopecia [3]. It mainly occurs in young middle-aged male patients and is seen more commonly in Africans as compared to Caucasians [4].

The etiology of FD is still unclear and not fully understood. In 1963, Bogg initially proposed that bacteria play a significant role in the onset of FD [5]. Staphylococcus aureus infections are stated to play a crucial role in the pathogenesis of FD, as they can be identified in nearly every case through culture from patients with FD [6,7]. The hypothesis suggests that cytotoxic proteins secreted by S. aureus, often found in active lesions, might function as superantigens that bind to major histocompatibility complex class II molecules, which implies the direct stimulation of T cells, resulting in the release of cytokines and follicular destruction without requiring processing by antigen-presenting cells [8,9]. An inherent abnormality of follicular orifices and abnormal immune response could make individuals more susceptible to the easy entry of microorganisms [8]. Reports of similar cases in family members support the role of genetic predisposition in the occurrence of FD [10,11]. Patients sometimes report the occurrence of FD following scalp injuries; however, the pathogenetic significance of this association remains unclear [12]. The initial manifestation of FD is characterized by primary follicular papules, which evolve into pustules, crusts, erythema, and follicular hyperkeratosis, and ultimately result in perifollicular scarring alopecia with follicular tufting. Diagnosis of FD relies on clinical observations, bacterial culture, and histopathological assessments [13].

## Case presentation

**Figure 1:**
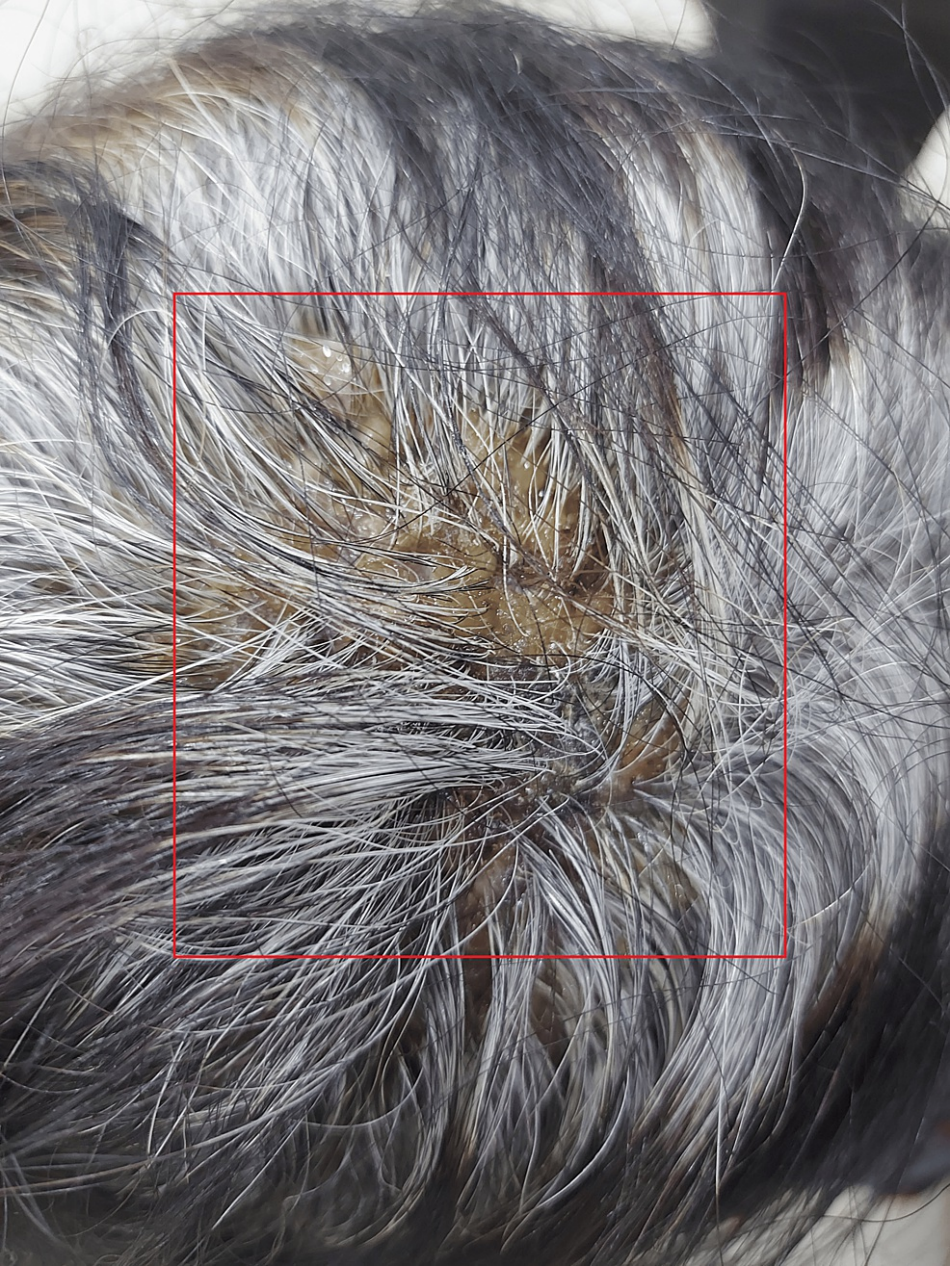
Red box showing the clinical presentation of folliculitis decalvans before treatment.

A 58-year-old female patient presented with a history of multiple coalescing pustules with pus discharge on the vertex region of the scalp for one year. The onset of symptoms was gradual, with the patient reporting the initial appearance of an erythematous follicular papule on the vertex and occipital area of the scalp. Over time, she experienced the development of follicular pustules and mild erythema, with yellow-gray scales, as shown in Figure 1.

**Figure 2:**
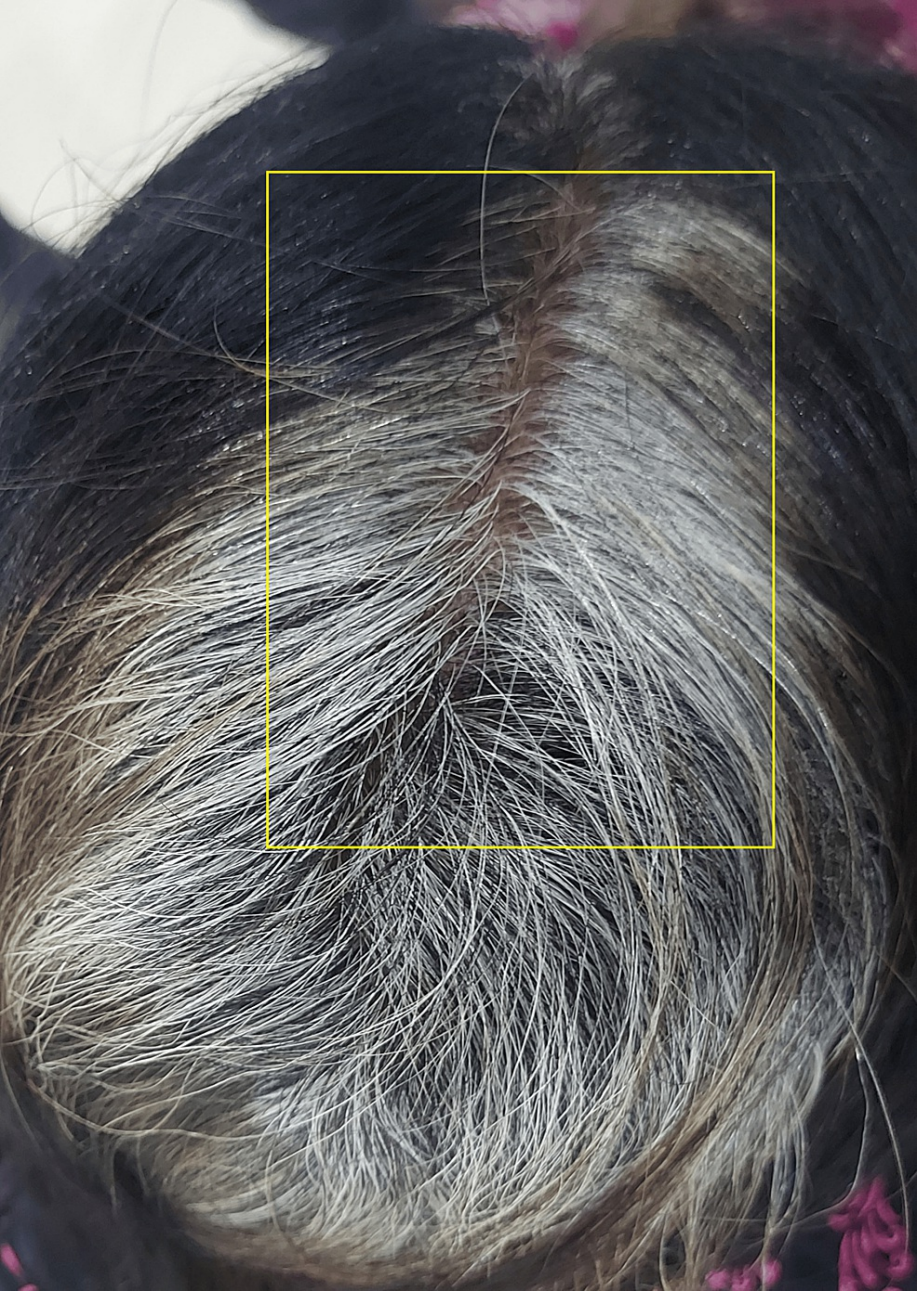
Yellow box showing posttreatment improvement after six months.

Tufted follicles were present, along with perifollicular hyperkeratosis, erosion, and hemorrhagic crusts. The patient complained of occasional mild pain, pus discharge, bleeding, itching, and a burning sensation. She reported no history of trauma, seborrheic dermatitis, or hair care treatment. She sought treatment from multiple doctors, receiving systemic and topical medications, but experienced no relief. Upon applying pressure to the perifollicular area, purulent discharge was observed. On clinical examination, multiple coalescent perifollicular pustular lesions were observed, with an accumulation of pus and crusting in the surrounding area. Tufting of hairs was also noted during the examination. The culture sample for laboratory testing was obtained using a sterile swab collected from intact pustules on the patient's scalp. The results of the culture analysis revealed a positive presence of S. aureus in the collected sample. Due to the active pus collection on the scalp, the dermoscopic examination was not feasible. Other blood investigations yielded results within normal limits. After clinical and laboratory investigations, a provisional diagnosis of FD was made. The patient was started on capsule isotretinoin 20 mg at night and capsule rifampicin 600 mg in the morning on an empty stomach daily. After six months of combination therapy, the patient showed a dramatic response, with complete resolution of lesions and normal hair growth observed on the vertex area of the scalp, as shown in Figure 2.

## Discussion

Effective management of patients with FD is crucial due to the potential for scarring alopecia and the risk of complete loss of hair follicles. Initiation of treatment is recommended during the active phase of the disease, characterized by the presence of indicators such as pustules, crusts, erythema, pruritus or pain, progressive scarring, and progressive hair loss. Conversely, once signs of inflammation are no longer present, the response to treatment tends to be less favorable. Therefore, timely intervention during the active phase is key to achieving better treatment outcomes in cases with FD.

The management of this disorder has posed significant challenges, with numerous published attempts using anti-inflammatory or anti-infective treatments. Success has been reported in selected cases employing various approaches, such as topical or intralesional administration of steroids, topical application of tacrolimus ointment, photodynamic therapy, and hair removal through shaving or neodymium-doped yttrium aluminum garnet (ND:YAG) laser [14-16]. Systemic treatments, including combinations such as zinc sulfate and oral/local fusidic acid, tyrosine, dapsone, infliximab, minocycline, rifampicin, clarithromycin, acitretin, isotretinoin, clindamycin, prednisolone, and notably the combination of rifampicin and clindamycin, have shown efficacy in certain instances [17-20]. The diverse range of treatments underscores the complexity of addressing this disorder, and individualized approaches may be necessary based on the specific characteristics of each case.

In this case, we tried a combination of oral isotretinoin and rifampicin, considering their inhibitory effects on the immune system and antibacterial action, respectively. Aksoy et al. analysis revealed that oral isotretinoin monotherapy led to a complete response in the majority of patients. They also proposed administering oral isotretinoin at a dose of ≥0.4 mg/kg/day for a minimum of ≥3 months to reduce the risk of relapse. Furthermore, the authors considered oral isotretinoin monotherapy as a promising treatment option for FD [21]. A retrospective study of different regimens by Tietze et al. found that nine out of 10 patients (90%) went into remission with isotretinoin monotherapy or in combinations with other antibacterial drugs after five to seven months of therapy as compared to other drugs like clindamycin, dapsone, and clarithromycin. They employed a range of diverse antibiotics initially, yielding effectiveness at the outset but demonstrating limited efficacy for long-term remission [22].

Jawade et al. showed dramatic remission with complete hair growth in a 26-year-old patient with FD after one month of therapy of oral isotretinoin (0.5 mg/kg), oral rifampicin, and topical fusidic acid cream [23]. Another case reported by Gemmeke and Wollina observed excellent response with complete remission and partial hair growth in a patient with FD after six-week treatment with a combination of capsule isotretinoin 40 mg daily, tablet clindamycin 300 mg/day, and tablet prednisolone 20 mg daily tapered within three weeks [24]. Various studies mentioned the role of rifampicin as monotherapy or in combination with other antibacterial and anti-inflammatory drugs in the management of FD, showing moderate to excellent responses with fewer chances of remission [8,13,16,22].

Apart from the impact on sebaceous gland lipid production inhibition, isotretinoin also exerts a direct inhibitory influence on the immune system. This includes a reduction in the expression of various pro-matrix metalloproteinases, hindering the migration of neutrophils into the skin [25]. Additionally, isotretinoin results in a decrease in the expression of toll-like receptor 2, a mediator of the immune response to Gram-positive bacteria [26]. Rifampicin is very effective against S. aureus, which stops its growth by inhibiting DNA-dependent RNA polymerase activity in bacterial cells [27].

From the aforementioned discussion, it can be inferred that a comprehensive strategy for addressing the root causes of FD involves isotretinoin's capacity to inhibit abnormal host immune response and inflammation, complemented by rifampicin's effectiveness in eliminating S. aureus.

## Conclusions

In conclusion, FD remains a challenging condition to manage due to its resistance to treatment and potential for scarring alopecia. This case highlights the potential of isotretinoin and rifampicin combination therapy in achieving favorable outcomes. Isotretinoin's ability to inhibit abnormal keratinization and inflammation, coupled with rifampicin's efficacy in eradicating S. aureus, offers a comprehensive approach to addressing the underlying factors contributing to FD.

FD remains a therapeutic challenge, necessitating a multidimensional approach. The presented case underscores the importance of timely intervention during the active phase of the disease, where signs of inflammation are prominent. Early intervention during the active phase of the disease is pivotal to prevent irreversible scarring. The multifaceted approach to treatment, encompassing antibacterial therapy, anti-inflammatory medications, and, in severe cases, immunosuppressive agents, reflects the diverse strategies employed to address the chronic and inflammatory nature of FD.
